# Radiotherapy in the Treatment of Unresectable Recurrent Tracheal Pleomorphic Adenoma: A Case Report and Review of the Literature

**DOI:** 10.7759/cureus.103786

**Published:** 2026-02-17

**Authors:** Sara Harbaj, Amina Majdi, Boutaina Agdi, Rania Chakir, Amine Lachgar, Karima Nouni, Hanan El Kacemi, Tayeb Kebdani, Khalid Hassouni

**Affiliations:** 1 Department of Radiotherapy, National Institute of Oncology, Faculty of Medicine and Pharmacy - University Mohammed V, Rabat, MAR

**Keywords:** inoperable tumor, multidisciplinary management, pleomorphic adenoma, recurrence, trachea, vmat

## Abstract

Pleomorphic adenoma of the trachea is a rare tumor, with fewer than 50 cases reported. Its nonspecific symptoms can delay diagnosis. We describe the case of a 48-year-old woman with recurrent obstructive pleomorphic adenoma of the trachea. She was initially treated with endoscopic resection in 2019. In 2024, she developed increasing respiratory distress due to an inoperable recurrence. The patient received volumetric modulated arc therapy (VMAT) with a dose of 20 Gy in five treatments of 4 Gy each. This provided rapid symptom relief, and significant regression was observed on radiological imaging at three and 12 months. Although complete surgical removal remains the best option, this case shows that radiation therapy can be an effective choice for inoperable or recurrent tumors. The positive clinical and radiological results we observed underscore the role of radiotherapy in achieving durable local control and symptom relief. This highlights the need for thorough evaluation by multiple specialists and long-term follow-up.

## Introduction

Pleomorphic adenoma is the most common benign tumor of the salivary glands, accounting for around 60-70% of tumors of the parotid gland [1]. It is a mixed tumor, combining epithelial and myoepithelial elements within a stroma that is often myxoid, chondromyxoid, or hyalinized, justifying its name pleomorphic adenoma or mixed tumor [2].

Their tracheal localization is exceptional, with fewer than 50 cases described in the literature [3]. Clinically, these tumors may remain asymptomatic for a long time or present with non-specific respiratory signs (dyspnea, stridor, cough), which may mimic asthma or pulmonary embolism [4,5]. 

Although endoscopy and imaging play a key role in tumor detection and assessment of local extension, definitive diagnosis relies on histopathological examination. Microscopic analysis of biopsy or resected specimens is essential to confirm pleomorphic adenoma and to differentiate it from other benign or malignant tracheal tumors.

Complete surgical excision remains the treatment of choice. However, when surgery is not feasible, radiotherapy (RT) may represent an alternative therapeutic option. We report here a case of obstructive recurrence of a tracheal pleomorphic adenoma treated with RT.

## Case presentation

We report the clinical course and pathologic features of a 48-year-old Moroccan woman who has been following up since 2019 for a pleomorphic adenoma of the trachea initially treated by endoscopic resection with placement of a prosthesis, removed in 2022. She has a history of pulmonary tuberculosis treated in 2020. In August 2023, the patient presented progressive high dyspnea, 12 kg weight loss, and general deterioration.

**Figure 1:**
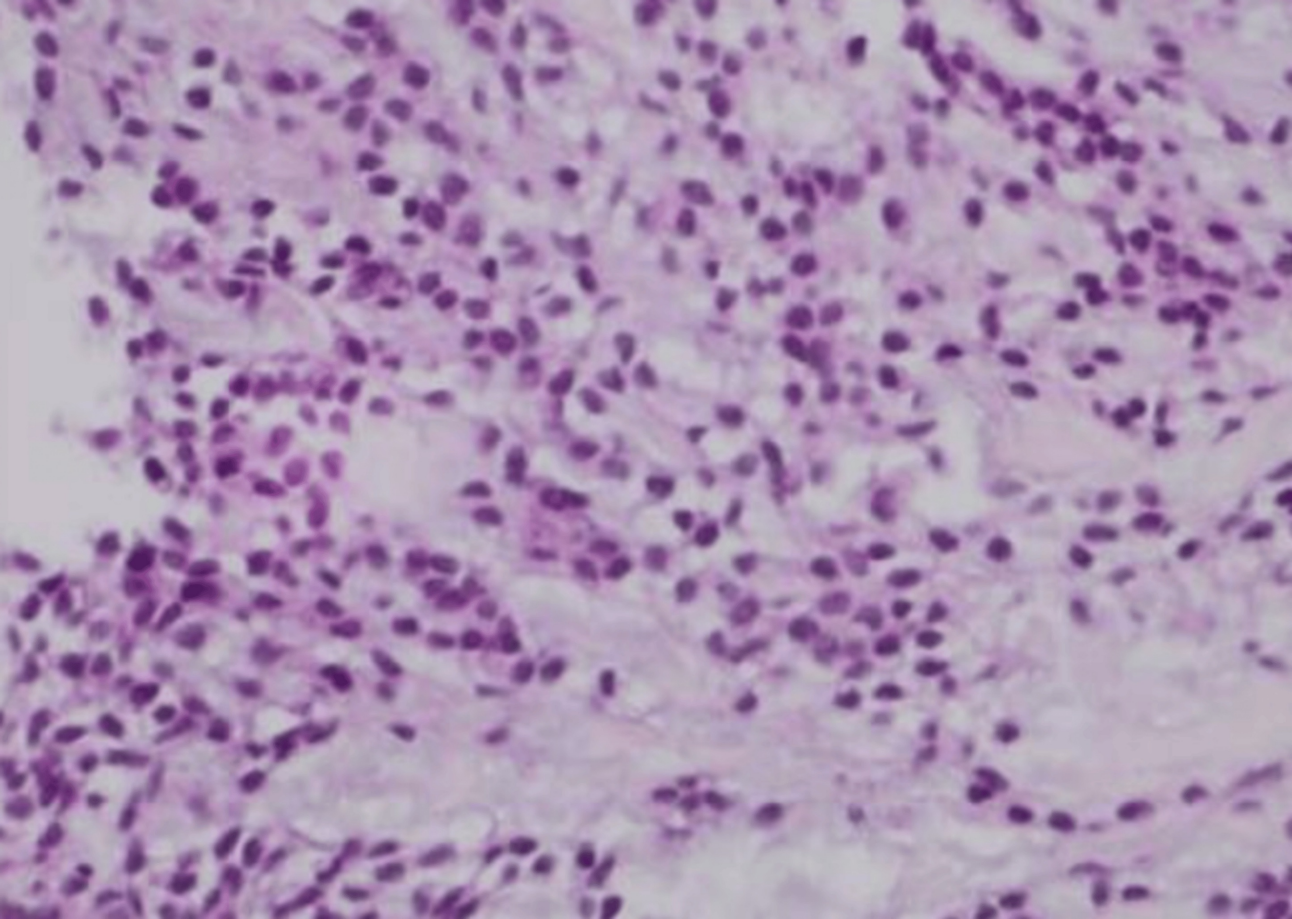
Histopathological examination of tracheal pleomorphic adenoma (hematoxylin and eosin staining, ×400).

Bronchial fibroscopy performed in 2019 enabled endoscopic biopsies, and histopathological examination confirmed the diagnosis of pleomorphic adenoma (Figure 1).

Microscopic examination demonstrated a well-circumscribed benign epithelial-myoepithelial tumor composed of duct-like epithelial structures and spindle-shaped myoepithelial cells embedded in a myxoid and chondromyxoid stroma, consistent with pleomorphic adenoma. No cellular atypia, mitotic activity, or features suggestive of malignant transformation were identified.

On May 20, 2024, bronchial fibroscopy revealed polypoid lesions invading the posterolateral right tracheal wall.

No repeat biopsy was performed at recurrence because the severity of airway obstruction made endoscopic sampling unsafe. Based on the patient’s prior histopathological findings, the absence of radiological features suggestive of aggressive malignant transformation, and the endoscopic appearance, the multidisciplinary team considered the lesion to be most consistent with recurrent pleomorphic adenoma rather than carcinoma ex pleomorphic adenoma (CXPA).

**Figure 2:**
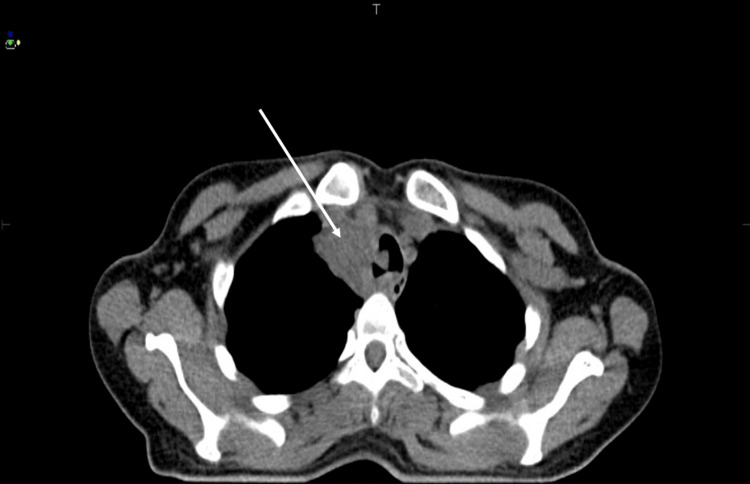
Axial CT image of the trachea showing the upper extent of the lesion. CT, computed tomography.

**Figure 3:**
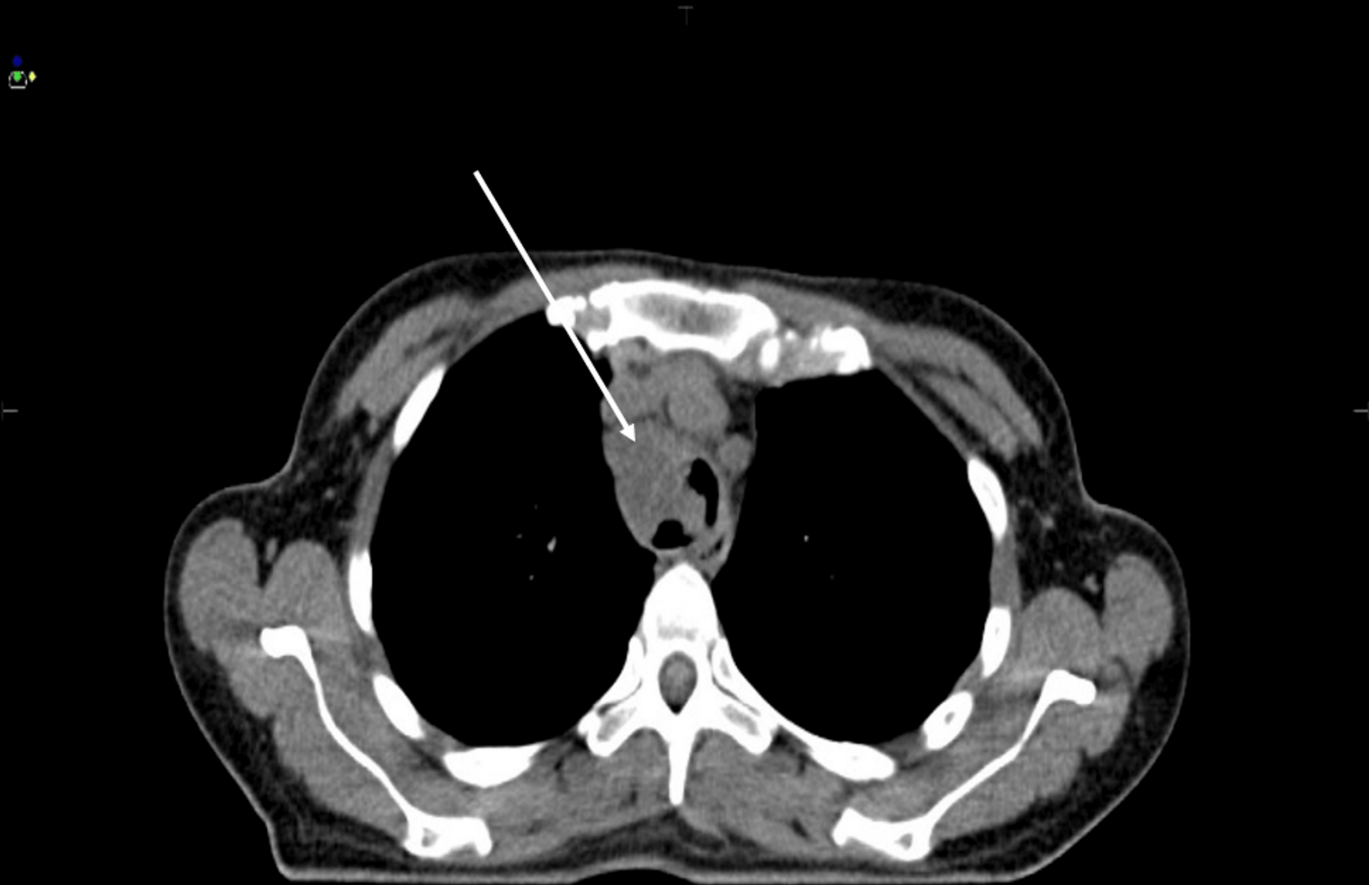
Axial CT image showing the lower extension of the tracheal lesion. CT, computed tomography.

Thoracoabdominal computed tomography (CT) performed on May 24, 2024, showed a poorly defined, almost circumferential tracheal mass measuring 78 mm in height and 28 mm in thickness, located 31 mm below the epiglottis. On axial sections, the lesion caused marked luminal narrowing of the trachea in its upper portion (Figure 2). The lower extension demonstrated close contact with surrounding mediastinal vascular structures, without a clear cleavage plane (Figure 3). Despite its locally extensive appearance, there were no radiological features suggestive of aggressive malignant behavior, such as distant invasion, necrosis, or lymph node involvement, and the imaging findings were considered more consistent with a benign tracheal tumor.

Presented at a thoracic multidisciplinary consultation meeting (RCP) on May 27, 2024, the tumor was deemed unresectable by thoracic surgeons due to its circumferential extension and involvement of adjacent mediastinal structures. RT was therefore selected with curative intent, aiming for durable local tumor control, based on published case reports demonstrating long-term control of unresectable tracheal pleomorphic adenomas treated with RT. However, the patient was initially unable to start treatment due to administrative delays despite multidisciplinary approval.

On June 23, 2024, she was urgently admitted with severe inspiratory dyspnea. On examination, she was polypneic, orthopneic, and diaphoretic, with an oxygen saturation of 78% on room air. Chest CT ruled out pulmonary embolism but confirmed a quasi-obstructive tracheal mass. Tracheostomy was considered but deemed unsafe due to the high tracheal location of the stenosis and the circumferential tumor infiltration with mediastinal extension.

The patient was admitted to the intensive care unit, where supportive medical management was initiated, including nebulized albuterol (Ventolin) and intravenous corticosteroids (methylprednisolone 120 mg/day). This treatment led to transient clinical stabilization but did not sufficiently relieve the critical airway obstruction, prompting the decision to proceed with emergency cytoreductive RT.

Emergency cytoreductive RT was administered, consisting of a total dose of 20 Gy administered once daily (OD) in five fractions of 4 Gy using the volumetric modulated arc therapy (VMAT) technique. This hypofractionated schedule was chosen to achieve rapid airway decompression and symptomatic relief in an unstable patient and was therefore delivered with palliative intent, rather than as definitive RT. Each RT session was administered in the presence of the attending physician and an intensive care specialist, under continuous oxygen therapy, to ensure close clinical monitoring during treatment. 

**Figure 4:**
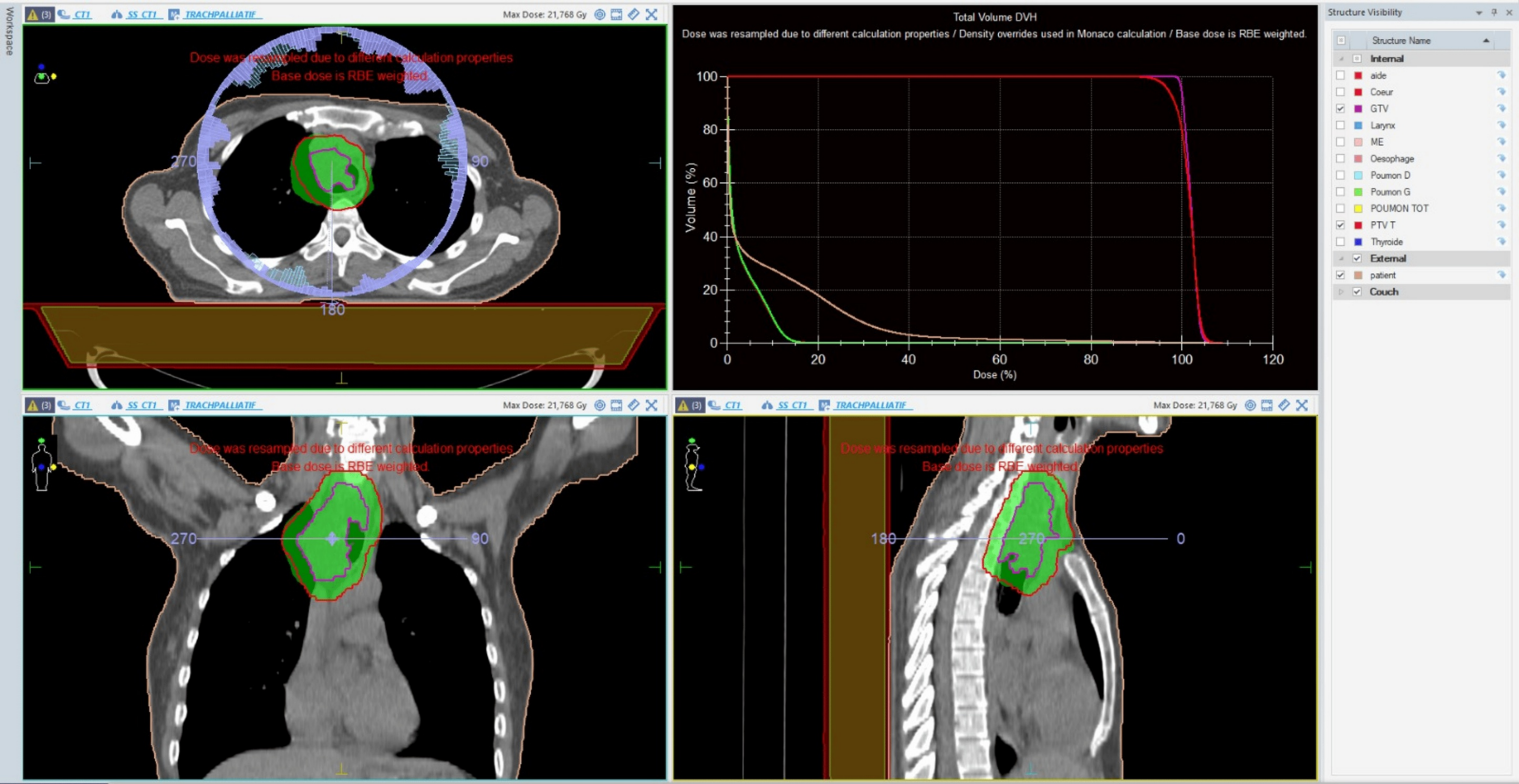
VMAT plan for an unresectable tracheal pleomorphic adenoma. Dosimetric images illustrating the radiotherapy plan. The prescribed dose was 20 Gy delivered in five fractions of 4 Gy. The GTV (purple) and the PTV (red) are displayed on axial, coronal, and sagittal views. VMAT, volumetric modulated arc therapy; GTV, gross tumor volume; PTV, planned target volume.

For RT planning, the gross tumor volume (GTV) was defined as the visible tracheal lesion on contrast-enhanced CT imaging. The clinical target volume (CTV) was generated by adding an appropriate margin to account for potential microscopic extension, and the planned target volume (PTV) was created to compensate for setup uncertainties and respiratory motion. Organs at risk (OARs), including the esophagus, spinal cord, lungs, and major mediastinal vessels, were contoured and respected according to institutional constraints (Figure 4).

The immediate outcome was marked by a clear improvement in ventilation, with a progressive reduction in oxygen requirements. At baseline, the patient presented with an oxygen saturation of 78% on room air and required 6-8 L/min of supplemental oxygen to maintain SpO₂ above 92%. She received intravenous methylprednisolone at a dose of 120 mg/day during the first 48 hours, followed by gradual tapering.

The patient remained hospitalized for 10 days after completion of RT under close clinical monitoring, until significant respiratory improvement was achieved and complete independence from supplemental oxygen was obtained.

A CT scan performed three months after completion of RT demonstrated a reduction in tumor size, measuring 65 × 32 × 27 mm, compared with the pre-RT dimensions of 78 × 28 × 31 mm. The evaluation scan performed one year after treatment showed a 30% reduction in tumor size compared with the pre-RT CT scan, with tumor response assessed according to RECIST 1.1 criteria, based on measurement of the longest axial diameter.

## Discussion

Pleomorphic adenoma of the trachea is a sporadic benign tumor of the lower respiratory tract. Fewer than 50 tracheobronchial cases have been reported to date, with only a minority arising exclusively from the trachea [3,5]. Its clinical presentation is often misleading, as symptoms such as dyspnea, stridor, or persistent cough may mimic asthma or chronic bronchitis, leading to delayed diagnosis [3,6,7]. In several cases, patients were treated for months with bronchodilators or corticosteroids before a definitive endoscopic diagnosis was established.

The standard treatment remains complete surgical excision, achieved through either segmental tracheal resection or endoscopic removal. When resection margins are tumor-free, surgery offers excellent long-term control and minimal recurrence [3,8,9]. Minimally invasive bronchoscopic procedures, such as laser therapy, argon plasma coagulation, or radiofrequency ablation, have been successfully employed in small endoluminal lesions or in patients unfit for surgery, providing rapid relief of airway obstruction and durable local control [8-10].

However, complete resection is not always feasible. In cases of extensive tracheal involvement, invasion of adjacent mediastinal structures, or major comorbidities, surgery may be contraindicated. In rare instances where surgical resection is not feasible, such as in cases of extensive tracheal involvement, mediastinal invasion, or major comorbidities, RT has been reported as a valuable alternative. Although experience remains limited, several case reports and retrospective analyses have demonstrated symptomatic improvement, local control, and prolonged survival in non-operable or recurrent pleomorphic adenomas treated with RT [3,11]. In addition to isolated tracheal cases, broader studies on salivary-gland pleomorphic adenomas have confirmed the role of postoperative or palliative RT in reducing recurrence and improving quality of life [11,12]. Recent observations also emphasize that RT can provide airway relief and meaningful clinical benefit when resection is contraindicated [3].

This observation supports the growing evidence that, although surgery remains the mainstay, RT may play a valuable role in carefully selected patients, particularly when airway obstruction or unresectability preclude surgical options.

Another important consideration is the potential for malignant transformation. Although pleomorphic adenoma is histologically benign, transformation into CXPA has been described, including at tracheal sites [10]. This highlights the importance of prolonged surveillance, combining clinical, radiological, and endoscopic evaluation, even after satisfactory local control.

The extreme rarity of tracheal pleomorphic adenoma, the lack of standardized management guidelines, and the variability of reported approaches underscore the need for a multidisciplinary decision-making process. Individualized treatment, balancing resectability, patient comorbidities, and available local expertise, remains essential to achieving optimal outcomes while minimizing morbidity.

In the literature, RT has predominantly been reported as an adjuvant or definitive treatment in selected cases of pleomorphic adenoma of the trachea, with conventional fractionation schedules delivering total doses of 50-66 Gy in 1.8-2 Gy fractions [7-13]. These regimens were generally administered to stable patients after incomplete resection or to patients with unresectable tumors without acute airway compromise.

In contrast, the present case represents an emergency clinical setting characterized by life-threatening airway obstruction, in which RT was delivered with palliative cytoreductive intent using a hypofractionated schedule to achieve rapid airway decompression. Such emergency use of RT has been rarely described in the literature, underscoring the need for individualized treatment strategies in critical presentations.

A review of the literature on pleomorphic adenomas of the trachea was conducted. All data are compiled in Table 1.

**Table 1:** Summary of reported cases of tracheal pleomorphic adenoma in the literature RT, radiotherapy; CXPA, carcinoma ex pleomorphic adenoma.

Authors (year)	Study type	Number of cases	Age/Sex	Tumor site	Treatment	Outcome
Liao et al., 2020 [3]	Case report	1	52, Female	Upper trachea	Surgical resection	No recurrence at 12 months
Takahashi et al., 2019 [4]	Case report	1	40, Male	Mid-trachea	Surgical resection	Symptoms resolved; no recurrence
Chen et al., 2023 [5]	Case series	7	7 cases (28–62 y)	Trachea and bronchi	Surgery (all)	All patients are alive; low recurrence rate
Inomata et al., 2023 [6]	Case report	1	44, Male	Lower trachea	Endoscopic resection	No recurrence at 6 months
Kushima et al., 2024 [7]	Case report	1	37, Female	Mid-trachea	Surgery + adjuvant RT (66 Gy)	No recurrence at 18 months
Sim et al., 2014 [8]	Case report	1	32, Female	Upper trachea	Endoscopic laser resection	No recurrence at 1 year
Gao et al., 2019 [10]	Case report	1	63, Male	Trachea	Surgery	Malignant transformation (CXPA) reported
Pomp et al, 1998 [13]	Case series	2	79, Female	Upper trachea (level of the fifth ring)	Excision through rigid bronchoscopy + Radiotherapy 50 Gy	No recurrence at 12 months
			58, Female	Upper trachea (below the larynx)	Excision via tracheotomy	Alive with no evidence of recurrence at 12 months
Current case (2025)	Case report	1	48, Female	Mid-trachea	Endoscopic surgery (2022) + RT for recurrence (20 Gy/5 fx, 2024)	Alive, no recurrence at 18 months

## Conclusions

RT may be considered as a therapeutic option in non-operable or recurrent tracheal pleomorphic adenoma. Beyond symptomatic relief, it can provide meaningful local control and improve respiratory function when surgery is contraindicated.

The observed clinical response supports its role as a feasible and well-tolerated alternative in selected cases. Management must remain multidisciplinary, with vigilant follow-up to detect any recurrence or malignant transformation at an early stage.
